# The Transferability and Design of Commercial Printer Settings in PLA/PBAT Fused Filament Fabrication

**DOI:** 10.3390/polym12112573

**Published:** 2020-11-02

**Authors:** Sisi Wang, Dagmar R. D’hooge, Lode Daelemans, Hesheng Xia, Karen De Clerck, Ludwig Cardon

**Affiliations:** 1 Centre for Polymer and Material Technologies (CPMT), Department of Materials, Textiles and Chemical Engineering, Ghent University, Technologiepark 130, 9052 Zwijnaarde, Belgium; Sisi.Wang@UGent.be; 2 College of Engineering, Zhejiang Normal University, Jinhua 321004, China; 3 Centre for Textiles Science and Engineering (CTSE), Ghent University, Technologiepark 70A, 9052 Zwijnaarde, Belgium; Dagmar.Dhooge@UGent.be (D.R.D.); Lode.Daelemans@UGent.be (L.D.); Karen.DeClerck@UGent.be (K.D.C.); 4 Laboratory for Chemical Technology (LCT), Department of Materials, Textiles and Chemical Engineering, Ghent University, Technologiepark 125, 9052 Zwijnaarde, Belgium; 5 State Key Laboratory of Polymer Materials Engineering, Polymer Research Institute, Sichuan University, Chengdu 610017, China; xiahs@scu.edu.cn

**Keywords:** printing parameter, mechanical property, printer transferability

## Abstract

In many fused filament fabrication (FFF) processes, commercial printers are used, but rarely are printer settings transferred from one commercial printer to the other to give similar final tensile part performance. Here, we report such translation going from the Felix 3.0 to Prusa i3 MK3 printer by adjusting the flow rate and overlap of strands, utilizing an in-house developed blend of polylactic acid (PLA) and poly(butylene adipate-co-terephthalate) (PBAT). We perform a sensitivity analysis for the Prusa printer, covering variations in nozzle temperature, nozzle diameter, layer thickness, and printing speed (*T*_nozzle_, *d*_nozzle_, LT, and *v*_print_), aiming at minimizing anisotropy and improving interlayer bonding. Higher mass, larger width, and thickness are obtained with larger *d*_nozzle_, lower *v*_print_, higher LT, and higher *T*_nozzle_. A higher *v*_print_ results in less tensile strain at break, but it remains at a high strain value for samples printed with *d*_nozzle_ equal to 0.5 mm. *v*_print_ has no significant effect on the tensile modulus and tensile and impact strength of the samples. If LT is fixed, an increased *d*_nozzle_ is beneficial for the tensile strength, ductility, and impact strength of the printed sample due to better bonding from a wider raster structure, while an increased LT leads to deterioration of mechanical properties. If the ratio *d*_nozzle_/LT is greater than 2, a good tensile performance is obtained. An improved *T*_nozzle_ leads to a sufficient flow of material, contributing to the performance of the printed device. The considerations brought forward result in a deeper understanding of the FFF process and offer guidance about parameter selection. The optimal *d*_nozzle_/*v*_print_/LT/*T*_nozzle_ combination is 0.5 mm/120 mm s^−1^/0.15 mm/230 °C.

## 1. Introduction 

**Figure 1:**
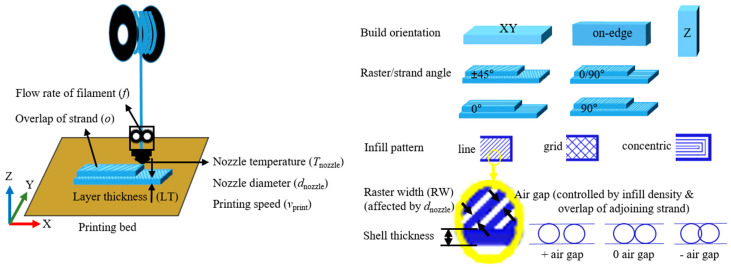
Principle of fused filament fabrication (FFF) alongside typical printer parameters.

Additive manufacturing (AM) has the potential to make complex shapes with less raw/scrap polymeric material by creating lightweight, topologically optimized structures compared to conventional manufacturing techniques [1,2,3]. Fused filament fabrication (FFF) involving polymers such as poly(lactic acid) (PLA) and acrylonitrile-butadiene-styrene polymer (ABS) is one of the well-known AM techniques. As shown in Figure 1, during FFF, a filament is pushed over a roller system to be melted and deposited layer by layer, explaining the equivalent term fused deposit modeling (FDM).

Both material properties and FFF conditions have an impact on the macroscopic properties of the final product, but the less studied (commercial) printer selection also affects the performance of FFF final material structures [4,5]. FFF design, however, is non-trivial [6,7], with, as is shown in Figure 1, many printing parameters such as build orientation [6,8,9], layer thickness (LT) [10], raster angle [11,12,13], raster width [14], nozzle diameter (*d*_nozzle_) [15,16], air gap [13,14], infill density and pattern [17,18], printing speed (*v*_print_) [10,19], nozzle temperature (*T*_nozzle_) [11,20,21,22,23], and feed rate [6,24]. On the material scale, insufficient bond strength between deposited filaments needs to be avoided; the interlayer strength in the building direction is often the weakest and most critical [13,14,15,16]. Adhesion is mainly driven by the thermal energy of the deposited molten polymer; once two strands are in contact with each other, the molecules can diffuse across the interface, leading to neck growth [25,26]. If the temperature is high enough, long entanglements can be formed through an inter-diffusion process, which increases the bond strength [27,28].

Most emphasis is currently on dimensional accuracy and control of the compressive, tensile, flexural, and/or impact strength, typically selecting one commercial printer and only a couple of printer parameters from Figure 1 as variables [6,11,19,21,29]. For example, Chacón et al. [6] demonstrated for the commercial SMARTFIL PLA filament that the *Z*-orientation sample showed an essential brittle fracture, as the force was perpendicular to the layer deposition direction, resulting in interlayer fusion bond failure. The *XY*-orientation sample exhibited a ductile fracture with considerable fiber deformation, resulting in multi-layer failure. Furthermore, Carlier et al. [10] demonstrated that increasing *v*_print_ (from 1 to 175 mm s^−1^) resulted in slightly lower tensile strength for flat *XY*-oriented printed PLA/poly(ethylene glycol) (PEG). Seppala et al. [22,23], in turn, reported that the most promising strategy to increase the weld strength is to increase *T*_nozzle_. Additionally, these authors mentioned a small increase in tear energy (mode III fracture) if *v*_print_ decreased. Christiyan et al. [19] found that tensile and flexural strength decreased with increased *v*_print_. Consistent with this observation, Tsouknidas et al. [17] reported that the tensile strength decreased (57 to 49 MPa) with increasing *v*_print_ (ranging from 30 to 220 mm s^−1^). Generally, *v*_print_ is consistent with the feeding speed [19,30] and, for a shorter deposition time, is also consistent with an increased *v*_print_, less interaction, and lower interlayer bonding between the contiguous raster results. In other words, *v*_print_ supports polymer diffusion to form a thicker weld. Pan et al. [31] analyzed the effects of *v*_print_ as well (30 to 60 mm s^−1^), and they surprisingly showed that the adhesion strength of the printed upstand cylinder using neat PLA increased with increasing *v*_print_ due to the minimization of material stacking and inner stress. Hence, it is still worthwhile to study the impact of *v*_print_, even in PLA-based systems.

A clear controversial parameter in the scope of FFF remains LT with reported results in various directions. Panda and co-workers [13] found that lower LT enhanced the tensile and flexural strength, whereas higher LT is good for the improvement of impact strength. They have demonstrated that a thick raster resulted in high temperature near the bonding surfaces which improved the diffusion and resulted in strong bond formation. Gomez-Gras et al. [18] found that a decreased LT favored neck growth and better cohesion among layers due to an increase in surface contact. Tymrak et al. [32] stated that a reduced LT (0.2–0.4 mm) led to a higher tensile strength, but they also reported that adhesion strength increased with increasing LT from 0.1 to 0.3 mm for a cylinder specimen [31]. This was attributed to slower heat losses and longer fused filament wetting results from the thicker sample slices such that the adhesive property was more effective. Sood et al. [14] observed with increased LT that the tensile strength first decreased and then increased. With increasing LT, fewer layers are required and the distortion effect is minimized, which is attributed to a less pronounced temperature gradient towards the bottom layers, implying increased strength. On the other hand, too large of an LT causes larger voids between printed lines, leading to weaker bonding. The raster/strand width is also controlled by *d*_nozzle_, which has also been put forward as a critical parameter next to LT. For example, Kuznetsov et al. [16] conducted a study using different *d*_nozzle_ (0.4, 0.6, and 0.8 mm) and assessed the tubular PLA sample strength using a three-point bending test with samples printed in an upright *Z*-position. The results suggested that increasing *d*_nozzle_ not only reduced the printing time but also increased the sample strength. Gomez-Gras et al. [18] stated that *d*_nozzle_ should be at least 1.5 times LT to ensure proper cohesion between filaments for enhanced part integrity. 

It should be further stressed that most FFF research emphasizes on single well-defined materials such as ABS [19,33] and (brittle) PLA [6,15,16,31], whereas only a few studies investigated hybrid compositions [10,34,35]. In any case, one focused on investigating the strength of the printed bars by tensile or bending tests, but few mentioned ductility, and almost all of them fixed the (commercial) printer type. 

Hence, the research field would benefit from more systematic studies covering all relevant printing and macroscopic parameters, addressing several commercial printer types and polymer blends. The present study therefore aims to assess the effect of various printing parameters on the final parts of FFF technology in a systematic manner, considering a predefined hybrid PLA/poly(butylene adipate-co-terephthalate) (PBAT) blend, investigating mechanical surface quality and efficiency behavior. PBAT was added due to its biodegradability and excellent ductility to obtain a biodegradable blend with balanced mechanical properties [34,36]. Specific attention was paid to the transferability of a given set of working commercial printer settings to another set for another commercial printer. It is shown that, after this translation, sufficiently homogenous samples with defined properties were produced by properly adjusting the processing parameters. 

## 2. Materials and Methods 

### 2.1. Materials

Polylactic acid (PLA) (Ingeo™ 3D850; abbreviated PLA3D with 0.5% d-isomer) was bought from Natureworks, Minnetonka, MN, USA. Poly(butylene adipate-co-terephthalate) (PBAT) with as brand name ecoflex F Blend C1200 was obtained from BASF, Ludwigshafen, Germany. It is an aliphatic-aromatic copolyester based on the comonomers 1,4-butanediol, adipic acid, and terephthalic acid. The polymers were dried at 50 °C overnight before processing. PBAT is added to PLA to obtain ductility as pure PLA is very brittle [34,36].

### 2.2. Filament Preparation and Printed Part Preparation with Reference Printer

The PLA/PBAT (mass ratio 80/20; [37,38]) filament was fabricated in our lab. PLA and PBAT pellets were compounded and granulated via a twin-screw extruder (Coperion ZSK18ML, Stuttgart, Germany) at a screw rate of 120 rpm with a temperature range from 160 to 210 °C. Then the compound was chopped into granules and fed into a single screw extruder (Brabender PL2000, Cologne, Germany) at 30 rpm with a temperature profile 180–210 °C from zone 1 to zone 4 (from the hopper to the die) to prepare filaments with an average diameter of 1.75 ± 0.05 mm. Afterwards, the filament was FFF processed into dumbbell (type 1BA, 74 mm × 5 mm × 2 mm, ISO527) and rectangle (100 mm × 10 mm × 4 mm, ISO 179) samples using the reference Felix 3.0 (IJsselstein, The Netherlands) printer first. 

The nozzle diameter (*d*_nozzle_) of the reference Felix printer was 0.35 mm and the samples were laid down flat (*XY*-oriented) on the printer bed covered with polyethyleneimine (PEI) film. The default printing parameter setup was designed as follows: shell thickness 1.05 mm, with a smooth shell to avoid premature cracking under load and to improve the strength of the sample; flow rate (*f*) 100% (generated by the software which is related to the input *v*_print_); infill overlap (*o*) 5%; lines infill pattern with raster angle ±45°; LT of 0.15 mm; first layer of 0.25 mm to confirm a good adhesion on the platform, thus the final thickness of 2.05 mm for a complete tensile bar (with first layer of 0.25 mm and 12 layers of LT 0.15 mm); printing speed (*v*_print_) 40 mm s^−1^ comparable to previous work [39]; nozzle and bed temperature (*T*_nozzle_ and *T*_bed_) of 210 °C and 50 °C.

### 2.3. Transferability for Two Commerical Printers

Comparable initial settings were used for the Prusa printer (i3 MK3; Prague, Czech). However, the extrusion force from the gear/roller changes for different printers, resulting in different flow during printing, thus affecting the bonding between strands and layers. This is caused by different hardware and control systems (e.g., variations in the frame, stepper motors, and extruder head) with different set points and proportionality constants [32]. Additionally, printers operate with software packages that interpret G-code according to different rules and algorithms. Each print software will generate different G-codes based on their default settings. Hence, for transferability, we focused on the comparison of the Felix and Prusa commercial printer toward a targeted printing performance, taking G-code from the same slicer (software repetier-Host for Felix printers V2.0.5 with Cura Engine embedded) to truly focus on the effect of intrinsic machine variations. 

The transferability was evaluated based on the tensile property of the specimen, realized by tuning the overlap rate and flow rate value, considering ranges as reported in Table 1 (variable (1) and (2)). After that, the effect of printing parameters (listed in Table 1, variables (3)–(6)) was considered with the Prusa printer and various settings consistent with Figure 1 and the discussion above. 

### 2.4. Characterization

#### 2.4.1. Mass and Dimension Variation

Mass measurements were done on an analytical balance (Precisa XR 2055M-DR, Dietikon, Switzerland) to evaluate the extrusion overall shape variation under different settings. The dimensions (length, width, and thickness) of the printed bars were measured after 2 days of storage using a caliper (Hogetex, Varsseveld, The Netherlands) to determine the dimension stability with different printing parameters. The results are the average of five measurements.

#### 2.4.2. Cross-Section Morphology

Microscopic observation of the internal structure of samples was performed to visualize the quality of the material deposition with different printing speed and nozzle diameter. The cross-sections after cryo-fracture and for a thin film (15 µm) cut from the tensile bars were observed using a polarizing microscope (POM) (Keyence VHX-1000, Mechelen, Belgium).

#### 2.4.3. Rheometric Analysis

Rotational rheometry analysis of PLA/PBAT was performed on a rotational rheometer (Anton Paar MCR702, Graz, Austria) equipped with parallel-plate geometry (diameter of 25 mm) in the shear range from 0.01 to 1000 s^−1^ at 210 and 230 °C under nitrogen atmosphere. The melt flow index (MFI) of the PLA/PBAT blend was tested regarding ISO 1133 with a load of 2.16 kg by the MFI test (Davenport, Hampshire, UK).

#### 2.4.4. Differential Scanning Calorimeter 

The selected material blend was subjected to different shear under various processing temperatures, nozzle diameters, and extrusion velocities, which may even lead to molecule breakage/degradation such that the crystallization temperature or crystallinity of the material is altered. DSC data were measured on a NETZSCH (214 Polyma^®^, Selb, Bavaria, Germany) instrument to investigate if these parameters affected the crystallization or not. Printed samples were heated from room temperature to 200 °C at 10 °C min^−1^ in a nitrogen atmosphere with a flow of 20 mL min^−1^, and the glass transition temperature (*T*_g_), cold crystallization peak (*T*_cc_), melt peak (*T*_m_), cold crystallization enthalpy (∆*H*_cc_), and melt enthalpy (∆*H*_m_) were recorded. The crystallinity of the sample was calculated according to:

(1) $$X_{c}=\left(\frac{∆H_{cc}+∆H_{m}}{∆H_{m}^{0}w_{PLA}}\right)\times 100\%$$

with ∆$H_{m}^{0}$ as the 100% crystalline polymer melt enthalpy (93 J g^−1^ [40] for PLA), and $w_{PLA}$ as the PLA mass ratio.

#### 2.4.5. Mechanical Analysis

The tensile properties of FFF produced bars were measured on an Instron 5565 machine (Norwood, MA, USA) with a load cell of 5 kN according to ISO 527. An extensometer 2620-603 Instron (Norwood, MA, USA) with gauge length 25 mm was used. A 1 mm min^−1^ tensile rate was applied until 0.3% strain was achieved to determine Young’s modulus. Afterward, a deformation at 10 mm min^−1^ was executed until the material broke, and yield stress (*σ*_Y_), maximum tensile stress (*σ*_M_), and tensile strain (*ε*) were subsequently recorded.

The three-point bending flexural test was performed with an Instron 4464 testing machine (Norwood, Massachusetts, USA) with a load cell of 2 kN according to standard ISO 178. The sample was placed on two supporting spans with a set distance of 64 mm, and a third loading pin was lowered at a constant rate (2 mm min^−1^) until a displacement of 15 mm was reached. The bottoms of all printed specimens faced downward in the tests.

Impact tests were conducted on a Tinius Olsen 503 Pendulum Impact Tester (Ulm, Baden-Württemberg, Germany) according to standard ISO 179. A V-notch was applied with a depth of 2 mm. The pendulum weight was 0.462 kg, which supplies nominal impact energy of 2.82 J and a released velocity of 3.46 m s^−1^. Tests were carried out after 2 days of conditioning in the standard atmosphere (23 °C; 50% humidity). At least 5 samples were tested to obtain an average.

## 3. Results

### 3.1. Evaluation of Transferability between Commercial Printer Felix and Prusa 

**Figure 2:**
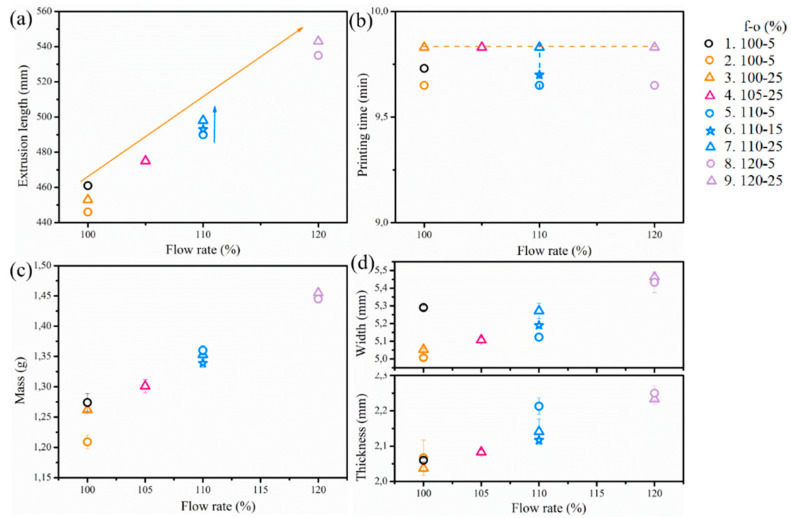
PLA/PBAT Prusa printed results with different (set) flow rate and overlap value (*f*-*o*, %): (**a**) software predicted extrusion length, (**b**) software predicted printing time, (**c**) mass, and (**d**) dimensions (Symbols in orange, pink, blue, and purple stand for flow rates of 100%, 105%, 100%, and 120%; the symbol of the circle, star, and triangle stand for overlap of 5%, 15%, and 25%.). Felix reference as black circles. Related results are listed in Table 2 and Table 3.

As shown in entries 1 and 2 in Table 2 and Table 3 with the same initial processing settings as Felix, the mass, dimensions, and tensile properties of bars printed by Prusa (entry 2) were slightly lower than the counterpart printed by Felix (entry 1). It was supposed that the forces from the gears in the two printers were different. To increase the strength of bars made by the Prusa printer, a negative gap was considered by tuning the overlap (*o*) value and flow rate (*f*) of the feeding material. The adopted *f*-*o* settings as well as the extrusion length and time generated by the software are listed in Table 2 (entries 3–9). The corresponding dimensional and tensile results are again listed in Table 3. A graphical representation is given in Figure 2, depicting the reference Felix results as black symbols.

It follows from Figure 2a that if the overlap value is fixed at 25% (triangle), with an increasing flow rate (100% to 120%) the extrusion length increased from 453 to 543 mm, which is animprovement by 20% as shown by the orange arrow in Figure 2a. The extrusion length increased only slightly from 446 to 453 mm (1.6% increase) with an increasing overlap value (5% to 25%) at a fixed flow rate 110%, as shown by the blue arrow in Figure 2a, resulting in a subtle increment in mass and dimensions correspondingly in Figure 2c,d. The printing time maintained constant with given *o* and varying *f* (e.g., orange dashed line in Figure 2b), which means that the set flow rate does not affect the line count and printing time. Overall, the longer extrusion length and higher extrusion volume coming out in the same printing time imply an increased real volumetric flow rate (*Q*_flow_, Table 2). In contrast to the flow rate, the line count and printing time increase slightly (e.g., blue dashed line in Figure 2b) with larger raster overlap at a given *f*. Hence, in Table 3, it is understandable that the mass and dimension values increased with increasing overlap and flow rate value, due to the larger amount of material extruded, as also shown in Figure 2c,d.

**Figure 3:**
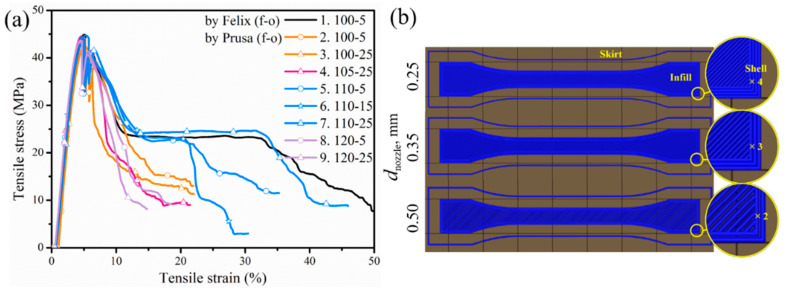
(**a**) PLA/PBAT Prusa printed with different flow rate and overlap (*f*-*o*, %); the orange, pink, blue, and purple curves stand for flow rates of 100%, 105%, 100%, and 120%, respectively; the circle, star, and triangle symbols stand for overlap of 5%, 15%, and 25%, respectively; at speed 40 mm s^−1^ and *d*_nozzle_ 0.35 mm. Felix reference curve in black. With *f*-*o* rate 110–25%, this curve can be approached; (**b**) diagram of strands/rasters in the tensile bar simulated with different nozzle diameter.

A higher amount of extruded material for the specimen means a stronger weld between printed lines so that tensile bars with better performance are expected. The stress/strain curves of the samples are illustrated in Figure 3a. In Figure 3, the black curve of PLA/PBAT material printed by Felix is displayed as a reference as well (entry 1 in Table 2). Concerning PLA/PBAT printed via Prusa, the samples printed with 100% and 105% flow rates (orange and pink curve in Figure 3a) failed more abruptly without a region of extended yielding under load due to insufficient filling, and the yield stress and maximal stress were lower than the sample printed via Felix. If the flow rate reached 110%, the failed samples were more ductile and have a longer region of plastic deformation (necking). Furthermore, the overlap value is important for the tensile property with the tensile strain becoming larger with increasing overlap since a negative gap enhances the interlayer bonding [13,14,21]. The tensile behavior of the Prusa sample with *f*-*o* of 110–25% is thus understandably close to that of the bar printed by the Felix printer as the blue curve with the triangle symbols (entry 7) showed the most similar tensile behavior compared to the Felix reference (black curve). This comparable tensile performance identifies the transferability between the two printers. However, the tensile property did not improve with continued increasing extrusion volume (flow rate of 120%), due to higher inner stress in the bar caused by more polymer melt being compressed during printing. The transformation from the Felix printer to the Prusa printer succeeds therefore with the *f-o* value 110–25%, which is the *f-o* setting applied for the subsequent sensitivity analysis considering Prusa printings. It can be expected that such transferability also works with other polymeric materials as well since condensed bars are the overall objective.

### 3.2. Sensitivity Analysis for Prusa Printer after Transferibility Based on Tensile Strenght

#### 3.2.1. Slicer Predictions

**Figure 4:**
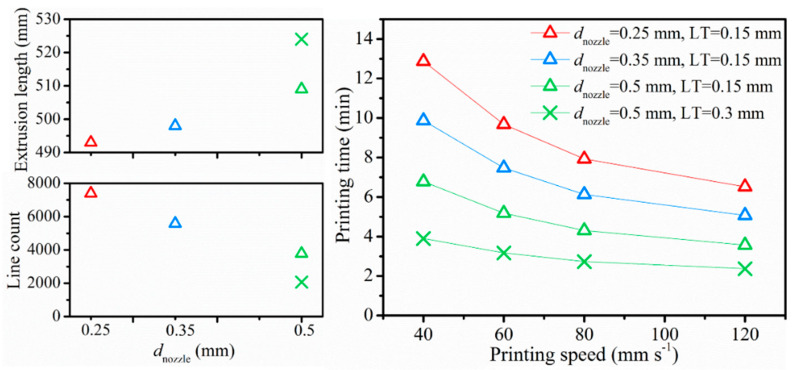
Data simulated by the slicer for the Prusa printer regarding different processing variations in Table 1. Raw data of actual selection combinations are listed in Table 4.

In what follows, the material was printed using Prusa with a fixed flow rate-overlap (*f-o*) value of 110–25% based on the previous results. The other printing parameters *d*_nozzle_, *v*_print_, LT, and *T*_nozzle_ were varied in the context of a sensitivity analysis, with the parameter variation range shown in Table 1. The nozzle size varies from 0.25 to 0.5 mm, *v*_print_ changes from 40 mm s^−1^ to triple of that, and LT has a default value of 0.15 mm with a larger LT of 0.3 mm only applied for *d*_nozzle_ equal to 0.5 mm. Focus is first on slicer predicts as practically it gives an intuitive prediction about printing time, extrusion volume, etc. Many end users consider this approach toward tuning parameters. The schematic diagram of the lay-down route given by the slicer is previewed in Figure 3b to show the inner structure of a printed sample. It was noticeable that the deposition routes differed from each other, as determined by *d*_nozzle_, since a wider raster width (RW) was produced with a larger *d*_nozzle_. Since a wider raster was applied, the number of extruded perimeter shells (composing the outermost edge of a part) decreased from 4 to 2 with a larger *d*_nozzle_, which also affected the infill section. Fewer lines were required, leading to a shorter time to accomplish a printing, as shown in Figure 4 (right part).

As visualized in Figure 4 (right part), the (predicted) printing time was also determined by *v*_print_ and LT, with a higher *v*_print_ and LT shortening the printing time, highly improving the production efficiency, and strongly reducing the manufacturing costs. The associated simulated slicer results were provided in columns 5–8 of Table 4. A minor increment of 3.2% was observed in extrusion length if *d*_nozzle_ increased from 0.25 mm to 0.5 mm. The (simulated) *Q*_flow_ was affected by the combined factors as calculated and mentioned in Table 4. Concluding, larger *d*_nozzle_, *v*_print_, and LT lead to shorter printing time, which means higher printing efficiency. 

#### 3.2.2. Dimension Stability and Morphology

**Figure 5:**
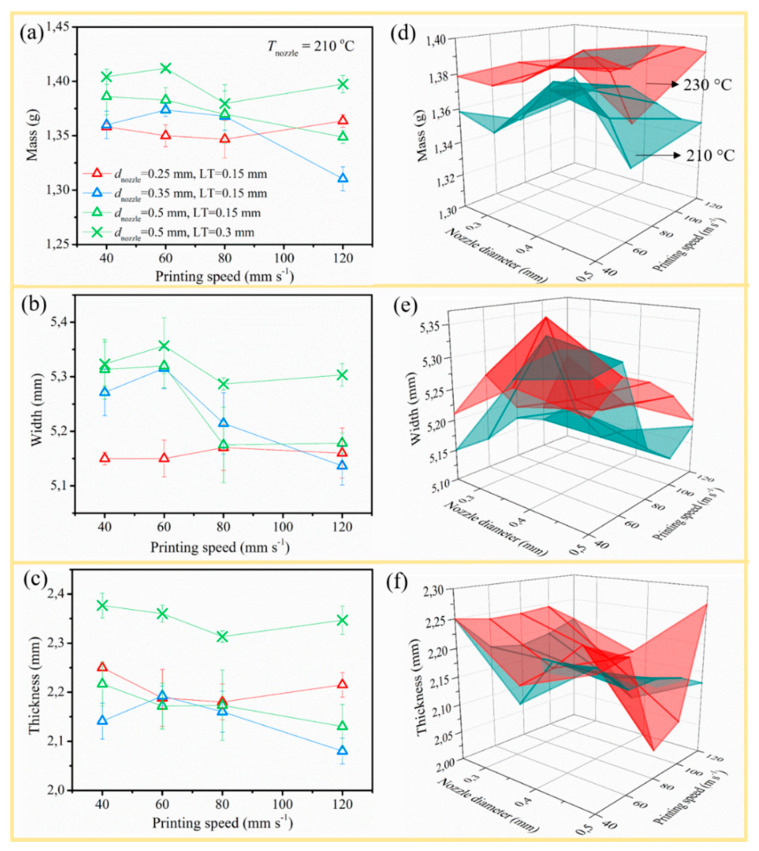
Effect of *d*_nozzle_ and *v*_print_ on (**a**) mass, (**b**) width, and (**c**) thickness of PLA/PBAT printed at 210 °C and (**d**–**f**) comparison to the results of samples printed at 230 °C with fixed LT 0.15 mm. Combinations from Table 4 with tabulated data in Table 5.

Both mass and dimensions have been measured to acknowledge the effects of the different processing parameters on the dimension stability, as illustrated in Table 5 considering the combinations defined in Table 4. The specific effects of *d*_nozzle_, *v*_print_, and LT on the mass, width, and thickness of PLA/PBAT printed at 210 °C are described in Figure 5 (left side), whereas the influence of *T*_nozzle_ is compared in Figure 5 (right side). 

**Figure 6:**
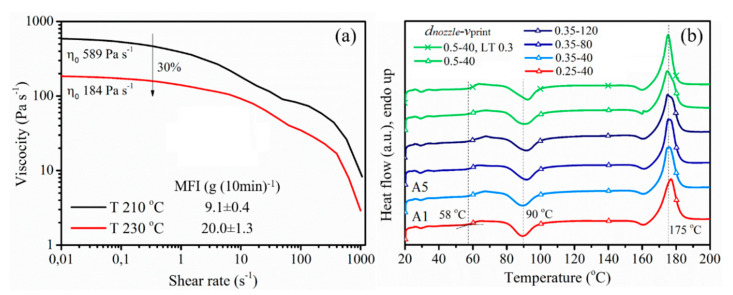
(**a**) Viscosity of PLA/PBAT as a function of shear rate and melt flow index (MFI) measured at different temperatures; (**b**) differential scanning calorimetry (DSC) curve of samples.

In Figure 5a, on average, the mass increased with increasing *d*_nozzle_ due to the larger amount of processed material, in accordance with previous research [16,18] and the discussion above (bigger extrusion length). If *d*_nozzle_ 0.35 and 0.5 mm were applied, the final mass dropped, correcting for experimental fluctuations with increasing *v*_print_, which can be clarified due to stretching of the molten strand. However, for a *d*_nozzle_ of 0.25 mm (red triangles line in Figure 5a), the mass difference with increasing *v*_print_ was insignificant, since more deposition time and heat transfer would trade the narrower RW off. In the right side of Figure 5, the sample mass at higher printing temperature (230 °C, red 3D surface) was significantly higher than the one printed at the lower temperature (210 °C, green 3D surface), considering the reduced viscosity below 230 °C (Figure 6a); thus, material flew through the nozzle more easily [39].

Concerning the effect of LT (comparison between the two green lines in Figure 5; left column), a higher LT resulted in larger mass because of a larger extrusion length pushed out, as simulated by the slicer software. A similar result was demonstrated by Carlier et al. [10] but, in contrast, Chacón et al. [6] indicated that the increment in LT has a negative influence on the sample mass, as a lower number of layers was needed to achieve the final structure. Therefore, printed devices with higher overall thickness need less material at first sight. In the present work, an increased LT was tuned toward a heavier sample because with seven layers of 0.3 mm LT, the final sample is 2.1 mm, which is only a bit higher than the final thickness of the other printed samples (2.05 mm). This higher mass was consistent with the bigger *L*_E_ given by the slicer.

Furthermore, the trend of width and thickness was similar to mass, as illustrated in Figure 5b,c. The dimensions increased with lower *v*_print_ and increased LT. The dimension difference was negligible with common LT. The nozzle diameter has little influence on the dimensions, considering the standard deviation. The result for *d*_nozzle_ 0.25 mm was an exception and a higher *T*_nozzle_ had a lifting effect on the width and thickness, consistent with a higher MFI. The individual effects were minor.

Since the material was subjected to higher shear rates with higher *Q*_flow_ and lower *T*_nozzle_, the molecular structure might change during printing, reflecting into a different crystallization behavior. However, there was no significant change in the DSC curve (Figure 6b), so polymeric materials showed almost the same *T*_g_, *T*_cc_ and *T*_m_ under various settings with a crystallinity of 23–26%. Therefore, it is put forward that these parameter variations do not lead to shear degradation or affect the crystallization of printed bars.

**Figure 7:**
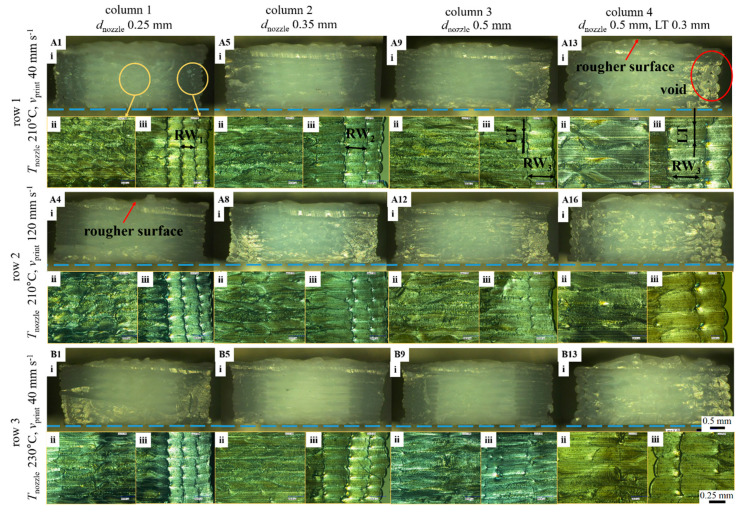
Polarized optical microscope (POM) pictures of the representative Prusa samples. Row 1, 2, and 3 stand for sample print at T_nozzle_ and v_print_ 210 °C and 40 mm s^−1^; 210 °C and 120 mm s^−1^; 230 °C and 40 mm s^−1^. Column 1–3 stand for d_nozzle_ 0.25–0.35–0.5 mm with LT 0.15 mm, column 4 stands for d_nozzle_ 0.5 mm with LT 0.3 mm; the sample codes are shown as defined. (ii) and (iii) are the enlarged view of the fracture surface (i) of the sample; the magnification is ×50 for (i), scale bar 0.5 mm; ×200 for (ii, iii), scale bar 0.25 mm.

The dimensional stability and inner void contribution had been identified by polarized optical microscope (POM) and the main results are shown in Figure 7. For columns 1 to 3 (Figure 7; A1, A5, and A9) in row 1 (default *v*_print_ of 40 mm s^−1^) at fixed LT 0.15 mm, no pronounced surface roughness was observed with increasing *d*_nozzle_. Following the schematic diagram in Figure 3b, a detailed zoom of the fracture surface of the tensile bar shows a larger RW in the shell part for a higher column number (refer to the enlarged iii of A1, A5, and A9). As the raster boundary cannot be clearly seen in the infill part (enlarged ii), the impact of voids inside the specimen can be excluded. In column 4 of Figure 7 (still row 1), a thicker layer (LT of 0.3 mm) was applied. Obvious voids can be seen in the perimeter part (A13, i), which may lead to premature cracking during a tensile test. 

Furthermore, row 2 showed images of samples printed at a higher speed (*v*_print_ 120 mm s^−1^). The nozzle movement may cause vibration, resulting in a rougher surface compared to row 1. In addition, samples printed at the higher temperature (Figure 7; row 3, *v*_print_ of 40 mm s^−1^) led to expansion due to sufficient melt flow with lower viscosity (Figure 6a). By observing all the fracture surfaces in Figure 7, PLA/PBAT specimen layers were in principle found to be bonded rather well during the selected printed conditions in such a way that the specimen appeared to be more like a homogeneous solid than a composition of individual extruded rasters. This implied at least on average a proper combination of nozzle size, filling speed, and printing temperature that created significant thermal bonding between both raster and layers causing greater fusing.

#### 3.2.3. Mechanical Performance

**Figure 8:**
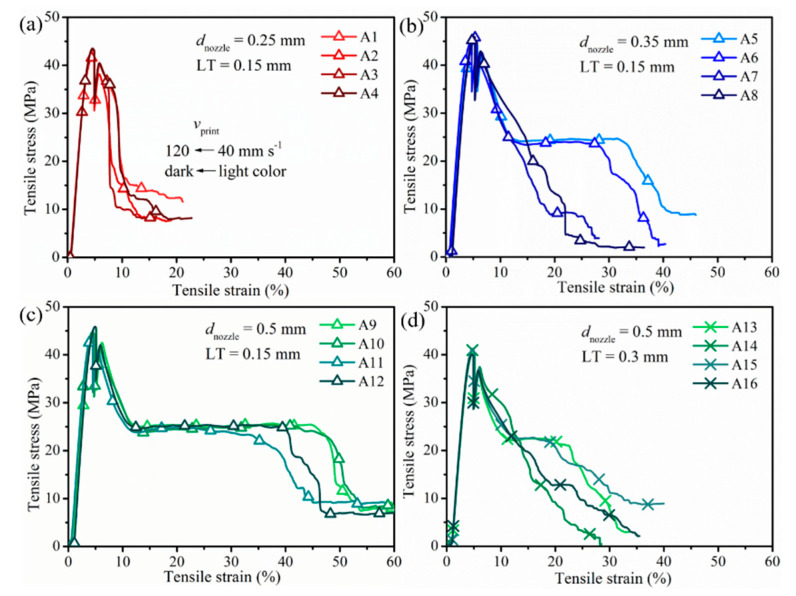
Comparison of tensile behavior of PLA/PBAT specimens printed with Prusa at 210°C with different *d*_nozzle_ (**a**) 0.25 mm, (**b**) 0.35, (**c**) 0.5 mm with LT of 0.15 mm, and (**d**) *d*_nozzle_ of 0.5 mm with LT equal to 0.3 mm (*v*_print_ speed was distinguished by gradient color from light to dark in each sample; legend refers to sample no. in Table 5.

**Figure 9:**
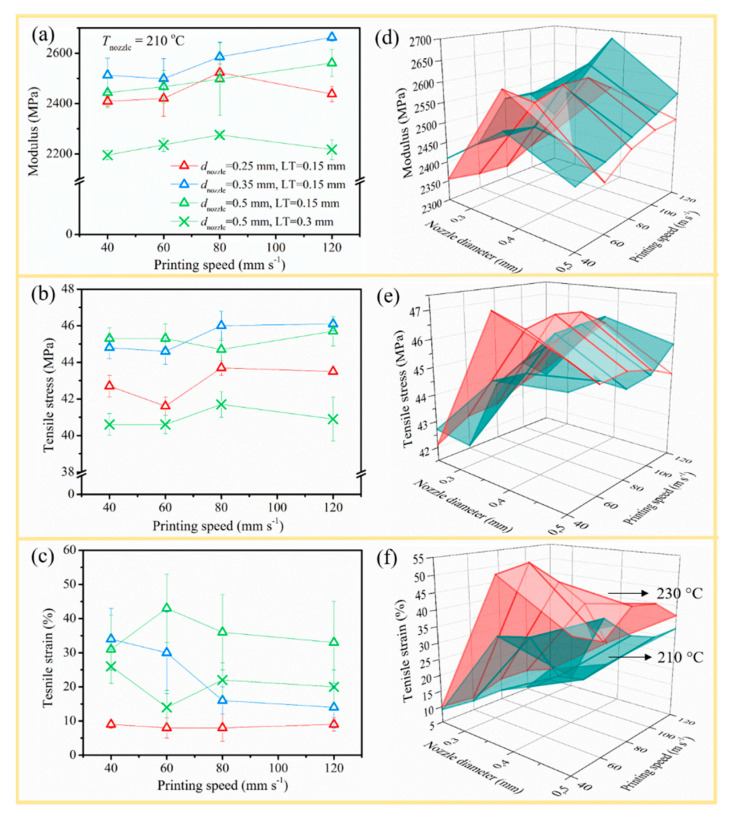
Effect of *d*_nozzle_ and *v*_print_ on (**a**) modulus, (**b**) maximum tensile stress, and (**c**) tensile strain of PLA/PBAT printed at 210 °C; (**d**–**f**) comparison to the results of samples printed at 230 °C with fixed LT 0.15 mm.

**Figure 10:**
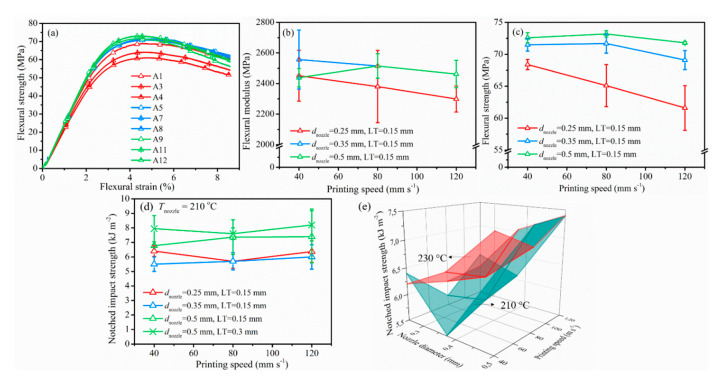
Flexural behavior of PLA/PBAT blend printed at 210 °C under three-point bending test (**a**) strain vs. strength curve; (**b**) flexural modulus; (**c**) flexural strength; (**d**) notched impact strength of PLA/PBAT blend with respect to various parameters; (**e**) further comparison to the results of samples printed at 230 °C with fixed LT 0.15 mm.

The aforementioned combinations of *d*_nozzle_, *v*_print_, LT, and *T*_nozzle_ were then employed to analyze the effect of processing parameters on the final part quality from a mechanical point of view. The tensile curves of interesting specimens were manifested in Figure 8 and the important indicators, Young’s modulus (*E*), maximum tensile stress (*σ*_M_), and tensile strain (*ε*), were drawn in Figure 9 with the corresponding tensile data accessible in Table 5 (right part). Quasi-static flexural mechanical properties were provided in Figure 10a–c. The notched impact strengths as functions of various parameters were depicted in Figure 10d. The flexural and impact tabulated results were displayed in Table 6. In what followed, the effects of each relevant variation were discussed. 

Figure 9a showed that for an LT of 0.15 mm and a *d*_nozzle_ of 0.25 mm (red triangles) with increasing *v*_print_, *E* first increased from 2409 to 2523 MPa, then decreased to 2438 MPa. For a *d*_nozzle_ of 0.35 and 0.5 mm (still LT of 0.15 mm; blue and green triangles), in contrast, *E* only increased with increasing *v*_print_. The *E* values for samples printed with *d*_nozzle_ of 0.35 mm (blue triangles) were the highest, highlighting the relevance of *d*_nozzle_ as the processing parameter. Tensile stress on average increased slightly (less than 3%) with larger *v*_print_ for all *d*_nozzle_, as shown in Figure 9b (still LT of 0.15 mm). For example, the tensile stress of samples with *d*_nozzle_ equal to 0.5 mm (45.3 ± 0.6 MPa; green triangle) was about 3 MPa higher (increase by 6%) than the result for *d*_nozzle_ equal to 0.25 mm (42.7 ± 0.6 MPa; red triangle) if *v*_print_ was fixed at 40 mm s^−1^, as a smaller nozzle can produce larger inner stress concentration during the longer printing time, thus producing lower tensile strength. A wider RW as produced by a larger nozzle implied that fewer lines were needed (Table 3), thus generating fewer voids between the adjacent lines [32].

In Figure 9c (still focus at LT of 0.15 mm), tensile strain increased from 14% to 34% (increase by 60%; blue triangle) with decreasing *v*_print_ if *d*_nozzle_ was 0.35 mm. This is because a lower *v*_print_ helps polymer diffusion to form a thicker weld, which benefits the mechanical strength of printed parts [22,23]. Similar results were given by Carlier et al. [10] and Christiyan et al. [19], who both reported that a higher deposition rate leads to a more porous structure due to a lack of the intra-layer adhesion during the process. However, the tensile strain kept at more or less the same level if a *d*_nozzle_ of 0.5 mm (green triangle) was used in spite of the increasing *v*_print_, owing to sufficient flow with such a larger diameter. Hence, processing parameters were interdependent, as highlighted in the introduction. It also followed from Figure 9c that the tensile strain improved significantly from only 9% to more than 30% with increasing *d*_nozzle_ for *v*_print_ equal to 40 mm s^−1^, benefiting from a better interlayer diffusion and negative air gap [14].

As shown in Figure 9 (green triangle vs. cross), LT was a crucial parameter. The modulus and the strain at break dropped dramatically when LT was increased to 0.3 mm. The tensile stress averages of PLA/PBAT decreased by 3 MPa (8% reduction) when LT was increased from 0.15 mm to 0.3 mm because bigger voids in the LT 0.3 mm sample festered the weld knot and strength, consistent with the POM picture in Figure 7 (column 4) and, for instance, the work of Tymrak et al. [32]. Furthermore, according to previous studies, the relation between LT and *d*_nozzle_ was an important indicator [16,18,24]. Gomez-Gras et al. [18] put forward that *d*_nozzle_ should be at least 1.5 times LT to ensure proper cohesion between filaments for enhanced part integrity. Kuznetsov and co-workers [16], in turn, demonstrated that the interlayer contact surface area was the most important factor to control the strength of the resulting part. They divided the ratio nozzle/layer thickness (N/L) into three zones based on experimental data and a curve-fitting approach. The cross-section of the single line looked rectangular if N/L was large and it resembled a circle of N/L was close to 1—thus, the larger the LT, the rounder the single line cross-section. The three zones were named as “unadvised”, “optimal”, and “rigorous”. With N/L between 1 and 2 (zone 1), the sample was claimed to break easily between layers so that it could only be used for low-duty prints. If N/L was between 2 and 4 (zone 2), optimal parts could be printed with good interlayer adhesion, showing bulk break under force. N/L above 4 (zone 3) was only recommended for the fabrication of crucial parts. These findings agreed well with our results, with samples printed with N/L 0.25/0.15 and 0.5/0.3 (N/L < 2) showing poorer tensile performance and samples with N/L 0.35/0.15 and 0.5/0.15 (2 < N/L < 4) displaying good ductility such that the material shows typical yielding and necking until fracture.

The flexural modulus and strengths from Figure 10a–c (LT of 0.15 mm) associated with *d*_nozzle_ equal to 0.35 and 0.5 mm (blue and green triangles) were typically much higher than those of samples printed with *d*_nozzle_ equal to 0.25 (red triangles), indicating a well-printed specimen with larger nozzle size. An increased LT almost did not change the flexural modulus and strength of the samples in the present study such that the results for a higher LT than 0.15 mm were not included in Figure 10a and Table 6. 

Furthermore, as shown in Figure 10d, in spite of the possible variation of *v*_print_, samples printed with a LT of 0.15 mm showed only higher notched impact strength with *d*_nozzle_ equal to 0.5 mm (green triangles vs. other triangles). This again showcased the better homogenous samples obtained with a larger nozzle at such LT; they experienced less shear and force during printing. A higher LT slightly improved the notched impact strength of the samples (green crosses vs. green triangles in Figure 10d), but at the same time, it negatively affected the tensile strength (not shown). The micro-voids of samples printed at larger LT (cf. last column in Figure 7) led to a zigzag fracture surface such that more energy could be more easily dissipated, therefore increasing the notched impact strength. From Figure 10e, it additionally followed that the deposition temperature influenced the adhesion between two successive layers and the bond quality, as larger notched impact strengths resulted from a higher printing temperature.

As is clear from the 3D surface comparisons in Figure 9d–f and Figure 10e, tensile and impact performance improved with increased printing temperature because a higher *T*_nozzle_ facilitated polymer diffusion to form a stronger weld with fewer voids, which was beneficial for the mechanical strength of the printed part [22,23,41,42]. The effect of *T*_nozzle_ on the flexural properties can, however, be neglected with only 20°C difference of printing temperature (not shown). 

## 4. Conclusions

In this research, we have studied the effect of nozzle diameter (*d*_nozzle_), printing speed (*v*_print_), layer thickness (LT), and processing temperature (*T*_nozzle_) on the dimensional stability and mechanical properties of ductile PLA/PBAT samples as obtained by FFF using the commercial printer Prusa i3 MK3.

Firstly, transferability from the commercial Felix 3.0 to the Prusa i3 MK3 printer was realized by adjusting the flow rate and overlap (*f-o*) to compensate for the different force applied by the feeding gear of the Prusa printer. Similar tensile performance of Felix bars under typical settings was achieved with an *f-o* value 110–25% on Prusa, compared to the *f-o* value of 100–5% on the Felix printer. The slicer predicted extrusion length depends on the flow rate, *f-o*, and *d*_nozzle_. It increased by 20% if the flow rate changed from 100% to 120%, slightly increased by 1.6% with overlap changing from 5% to 25%, and showed a minor increment of 3.2% when *d*_nozzle_ was increased from 0.25 mm to 0.5 mm. The mass of the printed bar showed a unanimous change, and the printing time was determined by both *v*_print_ and *d*_nozzle_, generating a wider raster with larger *d*_nozzle_. 

Secondly, different ranges of four main processing parameters were analyzed: *d*_nozzle_ (0.25, 0.35, 0.5 mm), *v*_print_ (40, 60, 80, 120 mm s^−1^), LT (0.15, 0.3 mm), and *T*_nozzle_ (210 °C, 230 °C). With increasing *d*_nozzle_, *v*_print_, LT, and *T*_nozzle_, samples with larger mass and dimensions were produced, and the sample surface became rougher, implying less dimensional stability. The printing time decreased with larger *d*_nozzle_, *v*_print_, and LT, which was good for efficient production. DSC results manifested that the shear stress during printing with various processing parameters did not cause molecular scale breakage or degradation. A larger *d*_nozzle_ was beneficial to obtain a negative air gap and better interlayer adhesion, and time-saving for efficient fabrication. Tensile modulus and tensile stress rose slightly with increasing *d*_nozzle_. While the tensile strain of PLA/PBAT sample declined with increasing *v*_print_ due to thinner line stretching, a higher *d*_nozzle_ compensated for the loss with stronger weld and bonding between lines such that the elongation of a sample printed with *d*_nozzle_ 0.5 mm remained constant. The flexural modulus and strength improved with larger *d*_nozzle_ and lower *v*_print_, showing the same trend with the tensile property. The notched impact strength improved slightly with *d*_nozzle_ in spite of other variations. It is worth noting that the strength and ductility of tensile bars decreased as LT increased. An optimal nozzle/layer thickness (N/L) ratio above 2 was raised for good quality samples. An elevated *T*_nozzle_ was beneficial for the tensile properties, resulting from sufficient flow and better interlayer diffusion during printing.

Overall, it followed that the sample performance was mainly affected by *d*_nozzle_ and LT while *v*_print_ and *T*_nozzle_ had lesser influence. Within the variation range of the current research, a combined low LT (0.15 mm), larger nozzle size (*d*_nozzle_ 0.5 mm), high printing speed (*v*_print_ 120 mm s^−1^), and high temperature (*T*_nozzle_ of 230 °C) were recommended for the optimal tensile property and reduced printing time. Ductility was well obtained if samples were printed with N/L ratio larger than 2. A larger nozzle and lower *v*_print_ were beneficial for the flexural property of the sample. The *d*_nozzle_ made difference in the notched impact strength of the specimens while other parameters had an insignificant influence on the impact strength.

## Figures and Tables

**Table 1:** Commercial printing parameters as introduced in Figure 1 and used in the present work; in bold, the default Felix settings. For the translation to Prusa, the variable settings.

Fixed Settings		Variable Settings for Translation	
Infill rate (%)	**100**	(1) Flow rate—*f* (%) ^1^	**100–**105–110–120
Shell thickness	**1.05**	(2) Overlap rate—*o* (%)	**5–**15–25
Strand orientation (°)	**±45**	(3) Printing speed—*v*_print_ (mm s^−1^)	**40–**60–80–120
Bed temperature (°C)	**50**	(4) Nozzle diameter—*d*_nozzle_ (mm)	0.25–**0.35–**0.5
Bed material	**PEI** ^2^	(5) Nozzle temperature—*T*_print_ (°C)	**210–**230
Nozzle material	**copper**	(6) Layer thickness—LT (mm)	**0.15**/0.3 ^3^

^1^ Volumetric flow velocity (*Q*_flow_) is 2.31–2.42–2.54–2.78 mm^3^ s^−1^ corresponding to the flow rate of 100–105–110–120% at *v*_print_ 40 mm s^−1^. ^2^ Polyethylenimine. ^3^ LT of 0.3 mm is only applied for bars printed with *d*_nozzle_ 0.5 mm, since LT should be smaller than 2/3 of *d*_nozzle_ [18].

**Table 2:** Extrusion length and time obtained with different flow rate and overlap settings as generated by software repetier-Host for Felix printers V2.0.5 with Cura Engine embedded; goal: PLA/PBAT printed at 210°C in a quest to obtain results similar to those of the Felix printer for the Prusa printer.

No.	Printer	*v*_print_ (mm s^−1^)	*f* (%)	*o* (%)	Extrusion Length, *L*_E_ (mm)	Line Count	Layer Count	Printing Time, *t*	Volumetric Flow Rate, *Q*_flow_, ^1^ (mm^3^ s^−1^)
1	Felix	40	100	5	461	5638	13	9 m 44 s	2.37
2	Prusa	40	100	5	446	5567	13	9 m 39 s	2.31
3	Prusa	40	100	25	453	5581	13	9 m 50 s	2.31
4	Prusa	40	105	25	475	5581	13	9 m 50 s	2.42
5	Prusa	40	110	5	490	5567	13	9 m 39 s	2.54
6	Prusa	40	110	15	493	5566	13	9 m 42 s	2.54
7	Prusa	40	110	25	498	5581	13	9 m 50 s	2.54
8	Prusa	40	120	5	535	5567	13	9 m 39 s	2.78
9	Prusa	40	120	25	543	5581	13	9 m 50 s	2.78

^1^$Q_{\mathrm{flow}}=\frac{\mathrm{flow}\;\mathrm{volume}}{t}={\pi r}^{2}L_{E}/t$ (2), *r* = filament diameter/2 = 1.75/2 mm = 0.875 mm.

**Table 3:** Corresponding actual dimensions and tensile properties for Table 2; entry 1 and 7: similar tensile behavior for the Felix and Prusa printers (see also Figure 3a).

No.	Mass (g)	Width ^1^ (mm)	Thickness ^1^ (mm)	Length ^1^ (mm)	E (GPa)	*σ*_Y_ (MPa)	*σ*_M_ (MPa)	*ε* (%)
1	1.27 ± 0.02	5.29 ± 0.01	2.06 ± 0.01	75.05 ± 0.26	2471 ± 110	37.2 ± 0.8	44.7 ± 3.7	38 ± 15
2	1.21 ± 0.01	5.01 ± 0.01	2.07 ± 0.05	74.97 ± 0.03	2456 ± 143	32.9 ± 0.1	43.2 ± 0.8	17 ± 2
3	1.26 ± 0.01	5.05 ± 0.01	2.04 ± 0.01	74.95 ± 0.03	2555 ± 44	32.6 ± 1.1	43. 2 ± 1.5	17 ± 1
4	1.30 ± 0.01	5.11 ± 0.01	2.08 ± 0.01	75.11 ± 0.03	2525 ± 72	35.1 ± 0.1	44.5 ± 0.1	14 ± 1
5	1.36 ± 0.01	5.12 ± 0.02	2.21 ± 0.02	74.99 ± 0.02	2573 ± 43	35.1 ± 0.4	44.1 ± 0.5	16 ± 8
6	1.34 ± 0.01	5.19 ± 0.01	2.12 ± 0.01	75.08 ± 0.02	2547 ± 55	35.3 ± 0.9	44.3 ± 0.3	20 ± 10
7	1.35 ± 0.01	5.27 ± 0.04	2.14 ± 0.04	75.12 ± 0.04	2513 ± 68	35.4 ± 1.4	44.8 ± 0.6	34 ± 9
8	1.45 ± 0.01	5.43 ± 0.06	2.25 ± 0.02	75.24 ± 0.03	2416 ± 105	35.3 ± 0.7	43.1 ± 0.3	10 ± 1
9	1.46 ± 0.01	5.46 ± 0.02	2.23 ± 0.02	75.27 ± 0.02	2359 ± 58	35.0 ± 0.4	43.0 ± 1.0	12 ± 1

^1^ The set width, thickness, and length (*W*_0_, *T*_0_, *L*_0_) for the tensile bar are 5, 2.05, and 75 mm, respectively. Here, *T*_0_ = 2.05 mm because the first layer thickness is set as 0.25 mm to obtain a good adhesion for the printing sample.

**Table 4:** Simulated printing values by slicer repetier-Cura Engine for Prusa-based printing.

No.	*d*_nozzle_ (mm)	LT (mm)	*v*_print_ (mm s^−1^)	Extrusion Length, *L*_E_ (mm)	Line Count	Layer Count	Printing Time, *t*	Volumetric Flow Rate, *Q*_flow_^1^ (mm^3^ s^−1^)
1	0.25	0.15	40	493	7395	13	12 m 52 s	1.92
2			60	493	7395	13	9 m 41 s	2.55
3			80	493	7395	13	7 m 56 s	3.11
4			120	493	7395	13	6 m 31 s	3.79
5	0.35	0.15	40	498	5581	13	9 m 50 s	2.54
6			60	498	5581	13	7 m 28 s	3.34
7			80	498	5581	13	6 m 8 s	4.07
8			120	498	5581	13	5 m 4 s	4.92
9	0.5	0.15	40	509	3779	13	6 m 47 s	3.76
10			60	509	3779	13	5 m 11 s	4.92
11			80	509	3779	13	4 m 18 s	5.93
12			120	509	3779	13	3 m 34 s	7.15
13	0.5	0.3	40	524	2069	7	3 m 54 s	6.73
14			60	524	2069	7	3 m 10 s	8.29
15			80	524	2069	7	2 m 44 s	9.60
16			120	524	2069	7	2 m 22 s	11.09

^1^$Q_{\mathrm{flow}}=\frac{\mathrm{flow}\;\mathrm{volume}}{t}={\pi r}^{2}L_{E}/t$ (2), *r* = filament diameter/2 = 1.75/2 mm = 0.875 mm.

**Table 5:** Mass, dimension, and tensile results of PLA/PBAT blend during sensitivity analysis.

Sample No.^1^	*v*_print_ (mm s^−1^)	Mass (g)	Dimension			Tensile Property			
			Width (mm)	Thickness (mm)	Length (mm)	E (MPa)	*σ*_Y_ (MPa)	*σ*_M_ (MPa)	*ε* (%)
	*d*_nozzle_ = 0.25 mm, LT = 0.15 mm, *T*_nozzle_ = 210 °C								
A1	40	1.36 ± 0.00	5.15 ± 0.01	2.25 ± 0.00	75.02 ± 0.01	2409 ± 18	34.1 ± 0.1	42.7 ± 0.6	9 ± 1
A2	60	1.35 ± 0.02	5.15 ± 0.03	2.19 ± 0.06	75.01 ± 0.03	2420 ± 72	32.8 ± 1.0	41.6 ± 0.5	8 ± 3
A3	80	1.35 ± 0.02	5.17 ± 0.04	2.18 ± 0.04	75.11 ± 0.04	2523 ± 34	34.7 ± 0.7	43.7 ± 0.4	8 ± 4
A4	120	1.36 ± 0.01	5.16 ± 0.05	2.21 ± 0.02	75.09 ± 0.04	2438 ± 31	35.0 ± 0.5	43.5 ± 0.0	9 ± 2
	*d*_nozzle_ = 0.35 mm, LT = 0.15 mm, *T*_nozzle_ = 210 °C								
A5	40	1.36 ± 0.01	5.27 ± 0.04	2.14 ± 0.04	75.12 ± 0.04	2513 ± 68	35.4 ± 1.4	44.8 ± 0.6	34 ± 9
A6	60	1.37 ± 0.01	5.32 ± 0.04	2.19 ± 0.02	75.51 ± 0.98	2499 ± 80	35.2 ± 1.2	44.6 ± 0.7	30 ± 12
A7	80	1.37 ± 0.01	5.21 ± 0.06	2.16 ± 0.04	75.11 ± 0.06	2586 ± 58	36.0 ± 0.9	46.0 ± 0.8	16 ± 4
A8	120	1.31 ± 0.01	5.14 ± 0.04	2.08 ± 0.03	75.17 ± 0.02	2664 ± 15	36.6 ± 0.3	46.1 ± 0.4	14 ± 6
	*d*_nozzle_ = 0.5 mm, LT = 0.15 mm, *T*_nozzle_ = 210 °C								
A9	40	1.39 ± 0.02	5.31 ± 0.05	2.22 ± 0.05	75.17 ± 0.02	2443 ± 61	36.2 ± 0.6	45.3 ± 0.6	31 ± 10
A10	60	1.38 ± 0.01	5.32 ± 0.04	2.17 ± 0.05	75.22 ± 0.02	2467 ± 64	36.5 ± 0.4	45.3 ± 0.8	43 ± 10
A11	80	1.37 ± 0.03	5.18 ± 0.07	2.17 ± 0.07	75.15 ± 0.04	2498 ± 144	35.9 ± 0.8	44.7 ± 0.6	36 ± 11
A12	120	1.35 ± 0.01	5.18 ± 0.02	2.13 ± 0.04	75.20 ± 0.04	2561 ± 54	35.9 ± 0.7	45.7 ± 0.8	33 ± 12
*d*_nozzle_ = 0.5 mm, LT = 0.3 mm, *T*_nozzle_ = 210 °C									
A13	40	1.40 ± 0.01	5.32 ± 0.04	2.38 ± 0.03	75.18 ± 0.04	2195 ± 18	33.5 ± 0.5	40.6 ± 0.6	26 ± 5
A14	60	1.41 ± 0.00	5.36 ± 0.05	2.36 ± 0.02	75.20 ± 0.07	2236 ± 26	33.6 ± 0.1	40.6 ± 0.5	14 ± 5
A15	80	1.38 ± 0.01	5.29 ± 0.01	2.31 ± 0.01	75.21 ± 0.01	2276 ± 13	34.1 ± 0.8	41.7 ± 0.7	22 ± 5
A16	120	1.40 ± 0.01	5.30 ± 0.02	2.35 ± 0.03	75.17 ± 0.04	2217 ± 39	34.2 ± 1.3	40.9 ± 1.2	20 ± 5
	*d*_nozzle_ = 0.25 mm, LT = 0.15 mm, *T*_nozzle_ = 230 °C								
B1	40	1.38 ± 0.01	5.21 ± 0.06	2.25 ± 0.04	75.09 ± 0.05	2354 ± 43	34.2 ± 0.4	42.1 ± 0.7	10 ± 2
B2	60	1.37 ± 0.00	5.26 ± 0.03	2.24 ± 0.03	75.12 ± 0.04	2339 ± 82	35.0 ± 0.7	42.8 ± 1.1	9 ± 1
B3	80	1.37 ± 0.02	5.19 ± 0.03	2.24 ± 0.03	75.09 ± 0.02	2331 ± 90	35.3 ± 0.2	42.9 ± 0.7	12 ± 4
B4	120	1.37 ± 0.01	5.26 ± 0.03	2.21 ± 0.04	75.17 ± 0.02	2387 ± 10	36.3 ± 0.4	43.8 ± 0.0	9 ± 2
	*d*_nozzle_ = 0.35 mm, LT = 0.15 mm, *T*_nozzle_ = 230 °C								
B5	40	1.39 ± 0.02	5.27 ± 0.10	2.17 ± 0.07	75.11 ± 0.08	2591 ± 105	36.5 ± 1.5	46.8 ± 0.8	49 ± 10
B6	60	1.38 ± 0.01	5.36 ± 0.02	2.18 ± 0.02	75.20 ± 0.04	2550 ± 79	35.9 ± 1.4	46.2 ± 1.6	51 ± 5
B7	80	1.39 ± 0.01	5.22 ± 0.08	2.22 ± 0.05	75.12 ± 0.08	2570 ± 58	36.6 ± 1.1	46.5 ± 0.9	44 ± 5
B8	120	1.34 ± 0.01	5.20 ± 0.02	2.16 ± 0.01	75.13 ± 0.07	2535 ± 39	36.0 ± 0.5	46.0 ± 0.6	21 ± 8
	*d*_nozzle_ = 0.5 mm, LT = 0.15 mm, *T*_nozzle_ = 230 °C								
B9	40	1.40 ± 0.04	5.26 ± 0.05	2.21 ± 0.04	75.15 ± 0.05	2510 ± 42	37.1 ± 1.1	46.2 ± 0.5	41 ± 8
B10	60	1.40 ± 0.05	5.30 ± 0.04	2.22 ± 0.03	75.18 ± 0.02	2421 ± 72	36.6 ± 0.4	45.2 ± 0.7	37 ± 4
B11	80	1.40 ± 0.00	5.28 ± 0.05	2.05 ± 0.21	75.17 ± 0.05	2470 ± 108	36.4 ± 0.6	45.3 ± 0.8	44 ± 9
B12	120	1.39 ± 0.02	5.19 ± 0.07	2.27 ± 0.09	75.13 ± 0.04	2495 ± 10	35.8 ± 0.7	44.6 ± 0.1	37 ± 1
	*d*_nozzle_ = 0.5 mm, LT = 0.3 mm, *T*_nozzle_ = 230 °C								
B13	40	1.44 ± 0.01	5.33 ± 0.05	2.40 ± 0.03	75.14 ± 0.07	2201 ± 52	33.9 ± 1.1	40.1 ± 1.2	19 ± 4
B14	60	1.39 ± 0.03	5.27 ± 0.06	2.37 ± 0.06	75.16 ± 0.03	2222 ± 10	34.1 ± 0.5	40.8 ± 0.5	23 ± 8
B15	80	1.43 ± 0.02	5.34 ± 0.04	2.38 ± 0.03	75.17 ± 0.06	2214 ± 61	35.1 ± 0.8	41.0 ± 0.6	16 ± 3
B16	120	1.42 ± 0.01	5.29 ± 0.03	2.39 ± 0.05	75.19 ± 0.10	2160 ± 35	34.2 ± 0.7	39.9 ± 0.5	21 ± 7

^1^ Link to Table 4; letter A and B stand for *T*_nozzle_ 210 and 230 °C, respectively.

**Table 6:** Notched impact strength and flexural properties of PLA/PBAT specimens at 210 °C with LT 0.15 mm. Sample names as introduced in Table 4.

Sample			Notched Impact	Flexural	
No.	*d*_nozzle_ (mm)	*v*_print_ (mm s^−1^)	Strength (kJ m^−2^)	Modulus, *E*_F_ (MPa)	Strength, *σ*_F_ (MPa)
A1	0.25	40	6.4 ± 0.4	2451 ± 166	68.4 ± 0.8
A3		80	5.7 ± 0.5	2380 ± 236	65.1 ± 3.3
A4		120	6.4 ± 0.7	2299 ± 85	61.6 ± 3.5
A5	0.35	40	5.5 ± 0.5	2557 ± 193	71.5 ± 1.0
A7		80	5.7 ± 0.6	2515 ± 80	71.7 ± 1.5
A8		120	6.0 ± 0.8	2462 ± 90	69.1 ± 1.5
A9	0.5	40	6.8 ± 0.1	2438 ± 60	72.6 ± 0.8
A11		80	7.4 ± 1.2	2515 ± 80	73.2 ± 0.5
A12		120	7.4 ± 1.7	2462 ± 90	71.8 ± 0.2
